# BeoNet-Halle – Aufbau einer multifunktionalen Datenbank zur automatisierten Extraktion von Versorgungsdaten aus Haus- und Facharztpraxen

**DOI:** 10.1007/s00103-023-03691-7

**Published:** 2023-04-20

**Authors:** Konstantin Moser, Rafael Mikolajczyk, Alexander Bauer, Daniel Tiller, Jan Christoph, Oliver Purschke, Sara Lena Lückmann, Thomas Frese

**Affiliations:** 1 Institut für Allgemeinmedizin, Medizinische Fakultät, Martin-Luther-Universität Halle-Wittenberg, Magdeburger Str. 8, 06112 Halle (Saale), Deutschland; 2 Institut für Medizinische Epidemiologie, Biometrie und Informatik, Medizinische Fakultät, Martin-Luther-Universität Halle-Wittenberg, 06112 Halle (Saale), Deutschland; 3 AG (Bio‑)Medical Data Science, Medizinische Fakultät, Martin-Luther-Universität Halle-Wittenberg, 06112 Halle (Saale), Deutschland; 4 Universitätsklinikum Halle, Datenintegrationszentrum SMITH Konsortium, 06120 Halle (Saale), Deutschland

**Keywords:** Ambulante Versorgung, Deutschland, Register, Versorgungsforschung, Elektronische Patientenakten, Outpatient care, Germany, Registries, Health services research, Electronic health records

## Abstract

Das Beobachtungspraxennetzwerk Halle (BeoNet-Halle) ist eine innovative Datenbank der ambulanten Versorgung, die seit 2020 deutschlandweit Versorgungsdaten von teilnehmenden Haus- und Facharztpraxen sammelt und für die Forschung und Versorgung zur Verfügung stellt. Aufgebaut und betreut wird die Datenbank vom Institut für Medizinische Epidemiologie, Biometrie und Informatik und vom Institut für Allgemeinmedizin der Medizinischen Fakultät der Martin-Luther-Universität Halle-Wittenberg. Weiterhin ist das Datenintegrationszentrum der Universitätsmedizin Halle an dem Projekt beteiligt. In die Datenbank sollen grundsätzlich anonymisierte und pseudonymisierte patientenbezogene Daten aus allen marktüblichen Praxisverwaltungssystemen einfließen. In diesem Artikel beschreiben wir den Aufbau und die Methoden der multifunktional nutzbaren Datenbank BeoNet und beziffern den aktuellen Datenbestand. Die Arbeitsabläufe von der Einholung, Übermittlung und Speicherung von breiten Einwilligungen (Broad Consent) werden dargestellt und Vorteile und Limitationen der Datenbank werden erörtert.

BeoNet-Halle enthält aktuell anonymisierte Datensätze von 73.043 Patienten aus 5 Arztpraxen. Es wurden 2.653.437 nach ICD-10 kodierte Diagnosen und 1.403.726 Verordnungen sowie 1.894.074 Laborergebnisse erfasst. Pseudonymisierte Daten von 481 Patienten wurden erfolgreich exportiert.

Das BeoNet-Halle ermöglicht eine nahezu lückenlose Darstellung des Versorgungsgeschehens in den teilnehmenden Praxen. Die Datenbank soll zukünftig praxisübergreifende Behandlungspfade der Patienten abbilden und qualitativ hochwertige Versorgungsdaten liefern, um zur gesundheitspolitischen Entscheidungsfindung und zur Optimierung von Versorgungsprozessen beitragen zu können.

**Zusatzmaterial online:**

Zusätzliche Informationen sind in der Online-Version dieses Artikels (10.1007/s00103-023-03691-7) enthalten.

## Abstract (en)

The Beobachtungspraxennetzwerk Halle (BeoNet-Halle) is an innovative database of outpatient care that has been collecting patient data from participating primary care and specialty practices throughout Germany since 2020 and making it available for research and care. The database is set up and maintained by the Institute of Medical Epidemiology, Biometrics and Informatics and the Institute of General Practice and Family Medicine of the Martin Luther University Halle-Wittenberg. Furthermore, the Data Integration Center of the University Medical Center Halle is involved in the project. In principle, anonymized and pseudonymized patient data from all commercially available practice management systems should flow into the databases.

In this article, we describe the structure and methods of the multi-purpose database BeoNet and quantify the current data stock. The workflow of collection, transfer, and storage of *broad consents* is described and advantages and limitations of the database are discussed.

BeoNet-Halle currently contains anonymized data of approximately 73,043 patients from five physician practices. Furthermore, it includes data from more than 2,653,437 ICD-10 diagnoses, 1,403,726 prescriptions, and 1,894,074 laboratory results. Pseudonymized data were successfully exported from 481 patients.

BeoNet-Halle enables an almost seamless representation of the care provided in the participating practices. In the future, the database will map patient treatment pathways across practices and provide high-quality care data to contribute to health policy decision-making and optimization of care processes.

## Hintergrund

Die epidemiologische Forschung mit Daten aus Hausarztpraxen entwickelte sich Mitte des 20. Jahrhunderts im Zuge der Arbeiten des englischen Hausarztes William Pickles. Er konnte aufzeigen, dass es möglich ist, mit der akribischen Sammlung von Informationen zu Krankheitsgeschichten von Patienten über viele Jahre hinweg wichtige Erkenntnisse zur Morbidität in lokalen Gemeinschaften zu gewinnen [1]. Mit der Entwicklung hin zur computergestützten Patientenversorgung veränderte sich diese Forschung auf Praxisebene wesentlich und es entstanden große ambulante Datenbanken mit longitudinalen Datensätzen von mehreren Millionen Patienten [2–6].

Solche Datenbanken haben ein hohes Potenzial für die Forschung und Versorgung, wie Studien aus anderen Ländern zeigen [6–9]. Zum Beispiel konnten Forscher aus dem Vereinigten Königreich basierend auf Daten der ambulanten Versorgung nachweisen, dass Krebsüberlebende ein mittel- bis langfristig erhöhtes Risiko für diverse Herz-Kreislauf-Erkrankungen gegenüber der Allgemeinbevölkerung aufweisen (mit großen Unterschieden zwischen den einzelnen Krebsentitäten; [9]). Eine weitere Studie identifizierte Risikofaktoren und frühe Anzeichen der Alzheimer-Krankheit, aus denen sich Präventionsmaßnahmen ableiten ließen. Hierfür wurden Langzeitbeobachtungen unter Verwendung verschiedener ambulanter Datenbanken zusammenführt [8]. Weitere Anwendungsmöglichkeiten der Datenbanken finden sich z. B. in der Arzneimittelsicherheit, der Deskription des ambulanten Versorgungsgeschehens oder der Entwicklung von Instrumenten zur Entscheidungsunterstützung [5, 7].

Für die Sekundärdatenanalyse werden in Deutschland bislang vorwiegend Daten von Sozialversicherungsträgern, allen voran Abrechnungsdaten der gesetzlichen Krankenversicherung verwendet [10]. Jüngste Bestrebungen zur Verbesserung der Datennutzung aus der Patientenversorgung sind die seit 2018 landesweit etablierten Konsortien im Rahmen der Medizininformatikinitiative (MII). Der Fokus dieser vom Bundesministerium für Bildung und Forschung (BMBF) geförderten Konsortien besteht u. a. im Aufbau und der Vernetzung von Datenintegrationszentren, die Voraussetzungen zum standortübergreifenden Datenaustausch zwischen Versorgern und der Forschung herstellen sollen [11]. Die deutschlandweite Etablierung von Netzwerken der Forschungspraxen begünstigt aktuell auch den Aufbau einer Datenbank der ambulanten Versorgung in Deutschland [12, 13].

Im Jahr 2020 wurden in Deutschland ca. 688 Mio. ambulante Behandlungsfälle von den privaten und gesetzlichen Krankenkassen gemeldet [14, 15]. Im gleichen Jahr zählte Deutschland laut Bundesärztekammer 161.400 ambulant tätige Ärzte. Jeder Behandlungsfall umfasst in der Regel eine Vielzahl an demografischen, abrechnungsrelevanten Angaben (Abrechnungsziffern und Diagnosen bzw. Beratungsergebnisse) und klinischen Angaben (Beratungsanlässe, Symptome, Untersuchungsbefunde, Laborergebnisse etc.). Bei dieser großen, klinisch relevanten Datenmenge kann man auch von einem „Datenschatz“ sprechen, der in jeder Arztpraxis vorliegt.

Die Extraktion und Verknüpfung von patientenbezogenen Daten aus Praxisverwaltungssystemen (PVS) stellt in Deutschland eine sowohl technische als auch datenschutzrechtliche Herausforderung dar. Mit Stand vom 31.09.2021 sind mindestens 132 verschiedene PVS auf dem Markt [16]. Um den Austausch kompletter Datensätze zwischen den verschiedenen PVS zu ermöglichen, wurde Anfang der 1990er-Jahre der Schnittstellenstandard „Behandlungsdatentransfer“ (BDT) vom Zentralinstitut der kassenärztlichen Versorgung entwickelt, der seitdem von den Softwareanbietern zum Austausch der Behandlungsdaten integriert wird [17]. Vorangegangene Studien zeigten jedoch auf, dass die automatisierte Datenextraktion mittels BDT-Schnittstelle mit großen Problemen einhergeht, weil diese von den Softwareanbietern unterschiedlich implementiert wird. Je nach PVS führt dies zu unterschiedlichen Problemen. Beispielsweise kann es vorkommen, dass Satzbeschreibungen einzelner BDT-Felder unterschiedlich interpretiert werden [18, 19]. BDT-Exporte liefern daher oftmals Ergebnisse, die nicht verwertbar sind [17].

Weiterhin ist für die Verarbeitung von sensiblen medizinischen Daten laut der Datenschutzgrundverordnung grundsätzlich eine Einwilligung der Patienten in die Nutzung ihrer Daten für medizinische Forschungszwecke vorgeschrieben [17]. Dies ist problematisch, weil bei ambulanten Datenbanken in der Regel viele unterschiedliche Patienteninformationen gesammelt werden und der Forschungszweck nicht immer von Beginn an definiert ist. Damit ein Patient nicht jedem einzelnen Forschungsvorhaben zustimmen muss, rücken daher zunehmend Patienteninformations- und Einwilligungsverfahren in den Fokus, die auf eine „breite Einwilligung“ (*Broad Consent*) ausgerichtet sind. Einen bundesweit konsentierten Mustertext hierzu liefert die MII [20]. Insbesondere im Krankenhaussektor wird zurzeit der Einsatz von *Broad Consent* als praktikabler Lösungsansatz verfolgt [11, 12, 21].

Neben strukturierten Daten, die zu einem gewissen Teil auch den Krankenkassen zugänglich sind, ist zunehmend auch die Informationsextraktion aus Freitextfeldern Gegenstand der Forschung, da Schätzungen zufolge unstrukturierte medizinische Daten mehr als 80 % der derzeit verfügbaren Daten in PVS ausmachen [22]. Dieser Datenschatz ist in Deutschland weitgehend unerforscht, da hierzulande kaum geeignete Instrumente existieren, mit denen sich solche Angaben ausreichend anonymisieren lassen. Mittels Merkmalsextraktion werden zurzeit erste Ansätze zur Aufbereitung von unstrukturierten medizinischen Daten erforscht [23, 24].

Das Ziel dieses Beitrags ist es, das Design und die Methoden vom BeoNet-Halle (Beobachtungspraxennetzwerk Halle) zu beschreiben, mit dem die in PVS verknüpften demografischen, abrechnungsrelevanten und klinischen Informationen aus teilnehmenden Haus- und Facharztpraxen exportiert, zusammengeführt und für weitere Analysen aufbereitet werden sollen. Weiterhin soll der aktuelle Stand der BeoNet-Datenbank am Beispiel einiger grundlegender Ergebnisse verdeutlicht werden.

## Das BeoNet-Halle

**Figure 1:**
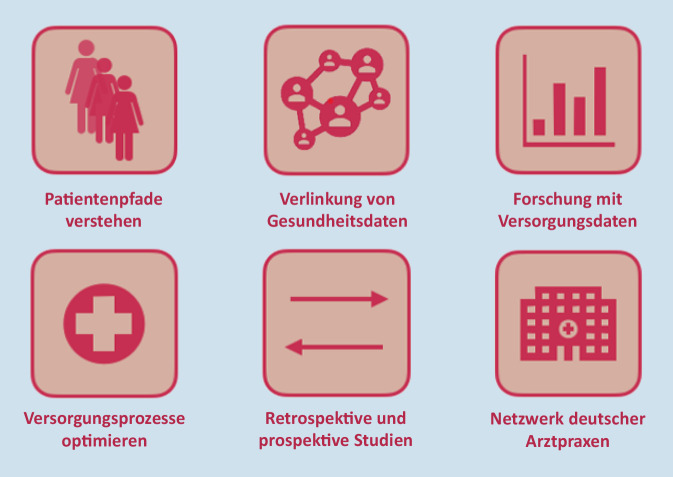

BeoNet-Halle ist ein Versorgungsforschungsprojekt, das seit 2018 von 2 Instituten der Medizinischen Fakultät der Martin-Luther-Universität Halle-Wittenberg aufgebaut und betreut wird: dem Institut für Medizinische Epidemiologie, Biometrie und Informatik und dem Institut für Allgemeinmedizin [25]. Das initiale Ziel des Projekts war es, eine ambulante Datenbank aufzubauen, die sämtliche dokumentierte Informationen aus PVS möglichst lückenlos erfasst, um somit longitudinale Krankheitsverläufe und systemische Interventionen abzubilden. Langfristig soll die BeoNet-Halle-Datenbank sektorenübergreifend mit anderen existierenden Datenquellen, wie z. B. stationären Behandlungsdaten, verknüpft werden, um Patientenpfade abzubilden (Verwendungsmöglichkeiten der Daten: Abb. 1).

Die Datenbank soll hochwertige Versorgungsdaten liefern und somit Versorgungsprozesse und die Versorgungsforschung unterstützen. Um diese Ziele zu erreichen, werden deutschlandweit Haus- und Facharztpraxen rekrutiert, die ihre Daten zur Verfügung stellen und Broad-Consent-Einwilligungen einholen. Das aktuelle BeoNet-Halle-Forschungsteam besteht aus Mitarbeitern der gründenden Institute sowie des Datenintegrationszentrums der Universitätsmedizin Halle. Das Team vereint verschiedene akademische Disziplinen, einschließlich der Allgemeinmedizin, Epidemiologie und Data Science.^1^ Zunächst wurde die Machbarkeit der Zusammenführung von patientenbezogenen Daten aus 2 unterschiedlichen PVS demonstriert. Das primäre Ziel war es, herauszufinden, ob eine Datenbank aufgebaut und die Daten aus teilnehmenden Praxen exportiert und in die Forschungsdatenbank überführt werden können. Hierfür wurden 2 Arztpraxen rekrutiert.

Zur Teilnahme am BeoNet-Halle unterzeichnen Arztpraxen eine Kooperationsvereinbarung zum Export von pseudonymisierten und/oder anonymisierten Daten. Das Exportmodul wird anschließend auf dem Primärsystem der teilnehmenden Praxis installiert. Der Export erfolgt auf Wunsch der teilnehmenden Arztpraxis manuell oder regelmäßig automatisiert (bspw. täglich). Parallel hierzu erhalten Mitarbeitende von Praxen, die dem pseudonymisierten Export zugestimmt haben, eine Unterweisung in die Einholung von Broad-Consent-Einwilligungen. Es wurde besonders darauf geachtet, dass der zusätzliche Aufwand beim Datenexport und dem Einholen von Einwilligungen für teilnehmende Praxen so gering wie möglich ist und dass sich die Abläufe optimal in den Praxisalltag integrieren lassen.

## Datenerhebung

**Figure 2:**
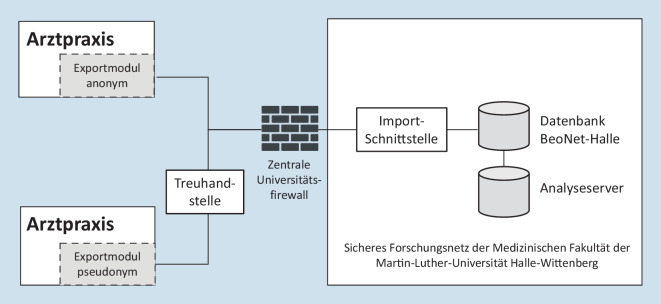

Unabhängig vom PVS der teilnehmenden Arztpraxis ist der Ablauf eines Datenexportes immer gleich:

1. Das Exportmodul prüft auf ausreichenden Speicherplatz der Festplatte.
2. Der Datenbankserver wird gestoppt.
3. Die benötigten Datenbankdateien werden in ein separates Verzeichnis kopiert.
4. Der Datenbankserver wird wieder gestartet.
5. Es erfolgt ein Durchlauf der Exportroutine mit der Datenbankkopie. Die Exportmodule konvertieren die Dateien immer gemäß der gültigen BeoNet-Halle-Tabellenstruktur und entsprechend dem Einwilligungs- oder Widerrufsstatus des Patienten anonymisiert bzw. pseudonymisiert (vgl. Tabelle Z1 im Onlinematerial).
6. Das Ergebnis der Konvertierung sind CSV-Dateien (durch Kommas getrennte Werte), die gesichert (per File Transfer Protocol) an die Importschnittstelle der Datenbank übermittelt werden, die sich innerhalb des Forschungsnetzes der Medizinischen Fakultät der Martin-Luther-Universität Halle-Wittenberg befindet (Abb. 2).

Falls ein Patient dem pseudonymisierten Export zugestimmt hat, werden die identifizierenden Daten (Name und Adresse) per sicherer Verbindung an die Treuhandstelle (THS) vom Datenintegrationszentrum (DIZ) der Universitätsmedizin Halle (UMH) übermittelt. Dort werden sie nach einem standardisierten Verfahren mit einem Pseudonym versehen, welches anschließend an die Datenbank transferiert wird. Einfache Pseudonyme (PID-1) werden von der THS für den Datenbankserver des BeoNet-Halle erzeugt. Eine doppelte Pseudonymisierung (PID-2) wird vorgenommen, wenn die Daten für Forscher für weitere Analysen aufbereitet werden.

Die Datenbank befindet sich innerhalb der kritischen Infrastruktur (KRITIS) der UMH, die besondere Anforderungen an die Informationssicherheit stellt und dem IT-Sicherheitsgesetz unterliegt. Nur der Datenmanager des BeoNet-Halle kann auf die Importschnittstelle und die sich darin befindenden unmittelbar exportierten Daten zugreifen. Die Importschnittstelle liegt auf einem virtuellen Fileserver in einem eigenen Netzwerk, bei dem die Zugangskontrolle gegeben ist. Somit kann nur der Datenmanager die Praxis identifizieren, aus der die Daten exportiert wurden. Damit wird die Vertraulichkeit gewahrt und Datenpakete, die aus einer Praxis stammen, können trotzdem entsprechend validiert werden. Für weitere Analysen werden Angaben, die eine Identifizierung der Praxis ermöglichen würden, entfernt. Innerhalb der Importschnittstelle werden Qualitätskontrollen durchgeführt, um herauszufinden, ob alle exportierten Felder anonymisiert wurden. Alle Mitarbeiter, die ggf. Zugang zu sensiblen Patientendaten haben, müssen zudem regelmäßig Informationssicherheitsschulungen absolvieren.

Um den vollständigen und fehlerfreien Export aller erforderlichen Daten gemäß der BeoNet-Halle-Tabellenstruktur sicherzustellen, wird je neuem PVS ein Test inkl. Dokumentation nach Erstellung eines Exportmoduls durchgeführt. Beim Ablaufen der Exportroutine werden zudem sogenannte Logfiles (Protokolldateien) erzeugt, auch um eventuelle Fehler nachzufolgen. Zudem werden sämtliche Aktivitäten der Importschnittstelle, wie z. B. eingehende Datenexporte, protokolliert. Die Anonymisierung wird sichergestellt, indem jedem Patienten ein nicht reversibler 35-stelliger Patientenschlüssel zugewiesen wird, zu dem kein Zuordnungsschlüssel existiert.

Die meisten Felder werden strukturiert exportiert, u. a. weil es für Abrechnungszwecke bestimmte Regeln gibt. Bei Freitextfeldern, die keinen Regeln unterliegen, werden die Feldlängen stark begrenzt. Weiterhin werden mit dem Einsatz einer Anonymisierungssoftware, die in das Exportmodul integriert ist, der Name des Patienten sowie weitere identifizierende Angaben sicher entfernt. Beim pseudonymisierten Export sollen zukünftig reine Freitextfelder exportiert werden. Dies bedarf allerdings einer Freigabe durch den Datenschutz, die aktuell noch nicht gegeben ist und weshalb ein Teil der Tabellen derzeit nicht exportiert wird. Wir sind bestrebt, die ambulanten Freitextinformationen zukünftig zu erschließen, und wollen dies bspw. durch das Ausschneiden potenziell identifizierender Merkmale (Parsing) weiter verbessern.

### Kontinuierliche Qualitätsverbesserung.

Sämtliche Prozesse (bspw. der Ablauf eines Datenexports oder der Rekrutierungsprozess) sind in Standard Operating Procedures (SOP) niedergeschrieben und unterliegen einem kontinuierlichen Verbesserungsprozess. Die Entwicklung, Installation und Aktualisierung der Exportmodule sowie die Verarbeitung von Daten sind vertraglich geregelt. Die Arztpraxis wird über sämtliche Abläufe rechtzeitig informiert und bei Bedarf unterstützt jemand den Export vor Ort. Alle Exporttermine werden vorab schriftlich fixiert.

## Verfahren für die Einholung, Übermittlung und Speicherung von Einwilligungen

Im Rahmen des Einwilligungsverfahrens wird angestrebt, von allen Patienten, die für einen Arztkontakt die Praxis aufsuchen, eine Einwilligung zur Nutzung der Daten zu bekommen. Für die Einwilligung haben wir uns an den bundesweit konsentierten Einwilligungsunterlagen „Broad Consent“ der MII orientiert und die Formulierungen im Wesentlichen übernommen. Die beim BeoNet-Halle verwendeten Einwilligungen haben ebenso keine Zweckbestimmung. Sämtliche Einwilligungsunterlagen wurden von der Ethikkommission und dem Justiziariat der Medizinischen Fakultät der Martin-Luther-Universität Halle-Wittenberg geprüft.

**Figure 3:**
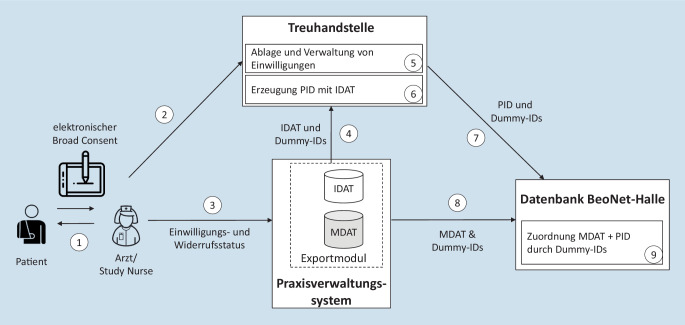

Von einem Arzt oder einer vom Arzt beauftragten Person wird dem Patienten zunächst eine Patienteninformation ausgehändigt und es werden offene Fragen geklärt. Anschließend wird dem Patienten die elektronische Einwilligungserklärung mittels Tablet zur Prüfung und Unterschrift übergeben (Abb. 3). Die Einwilligungen sind mit dem jeweiligen Praxisstempel versehen.

Der Einwilligungs- oder Widerrufsstatus des Patienten wird vom Praxispersonal in die elektronische Patientenkartei eingetragen. Die Eintragung erfolgt mit Hilfe von Kürzeln, die in regelmäßigem Abstand vom Exportmodul ausgelesen werden. Beispielsweise wird das Kürzel „beonet ja“ verwendet, wenn ein Patient der Nutzung seiner Daten zugestimmt hat. Die Karteikürzel für die Einträge werden pro Praxis jeweils schriftlich fixiert und müssen dann konsequent verwendet werden. Um Fehler bei der Eingabe der Kürzel zu vermeiden, erhalten ausgewählte Mitarbeiter der Praxis, die Einwilligungen einholen, eine umfangreiche Unterweisung in die korrekte Verwendung der Kürzel.

Der Patient kann weiterhin jederzeit und ohne Angabe von Gründen schriftlich oder mündlich seine Teilnahme in der Arztpraxis widerrufen. Sobald der Widerruf in der Praxis eingegangen ist, ist diese vertraglich dazu verpflichtet, das entsprechende Kürzel für den Widerruf in die Patientenakte einzutragen und die THS über den Widerruf zu informieren. Die THS muss sicherstellen, dass der Widerruf dem Wunsch des Patienten entsprechend erfolgt ist. Das Pseudonym wird an den Datenmanager zur Löschung aller Daten des Patienten weitergeleitet [26].

Die unterschriebenen Einwilligungen werden in eine PDF-Datei umgewandelt und automatisch per VPN (Virtual Private Network) an eine von anderen Projekten getrennt gehaltene Instanz innerhalb der THS des DIZ übermittelt. Dort werden sie zentral abgelegt, verwaltet und bspw. mit einem Pseudonym versehen. Von den Patienten, die eingewilligt haben, werden die identifizierenden Daten (IDAT) und ein vom Exportmodul erzeugtes temporäres Pseudonym („Dummy-ID“) an die THS übermittelt. Die Dummy-ID ist nur innerhalb der Praxis einem Patienten zuordenbar und dient zur späteren Verknüpfung von medizinischen Daten und dem von der THS erstellten dauerhaften Pseudonym. Die IDAT bestehen aus dem Namen und der Adresse des Patienten. In der THS werden aus den IDAT dauerhafte Pseudonyme mit dem zertifizierten Pseudonymisierungsinstrument gPAS der Universitätsmedizin Greifswald erzeugt [27]. Durch den Einsatz von gPAS soll langfristig eine Zusammenführung auch mit anderen Datenquellen erleichtert werden, bei denen das Instrument ebenfalls eingesetzt wird. Die medizinischen Daten (MDAT) und dazugehörige Dummy-IDs werden direkt an die Importschnittstelle der Datenbank übermittelt [25].

## Welche Daten werden gesammelt?

Tab. 1 zeigt eine Übersicht über die im BeoNet-Halle aktuell erfassten Variablen getrennt nach Exportart. Anonymisiert exportierte Felder umfassen demografische Patienteninformationen einschließlich Geburtsjahr und -monat, Geschlecht und Nationalität, administrative Angaben wie den Einwilligungsstatus und das Datum des Arztkontaktes, Body-Mass-Index (BMI), Rauchstatus und andere Risikofaktoren, Schwangerschaft und Anzahl der Kinder, nach ICD-10 (internationale statistische Klassifikation der Krankheiten und verwandter Gesundheitsprobleme) klassifizierte Diagnosen, Verschreibungsdetails einschließlich des Medikamentennamens und der ATC-Wirkstoffklasse^2^, klinische Parameter, wie bspw. Blutdruck und Gewicht, Laborergebnisse, sämtliche Abrechnungsziffern und z. B. Überweisungen. Bei der pseudonymisierten Exportvariante können strukturierte Angaben aus studienspezifischen Instrumenten wie Fragebögen extrahiert werden.

**Table 1:** 

Patientenangaben	Variablen
**Exportart anonym**	
*Demografische Angaben*	Geburtsjahr und -monat, erste 3 Stellen der Postleitzahl, Versichertenart, Geschlecht, Nationalität
*Administrative Angaben*	Datum Arztkontakt, Einwilligungsstatus
*Klinische Parameter*	Laborergebnisse, Blutdruck, Größe, Gewicht, Temperatur, Pulsrate
*Risikofaktoren*	Rauchstatus, Body-Mass-Index
*Schwangerschaft oder Kinder*	Anzahl Kinder, Schwangerschaftsstatus
*Diagnosen*	(Dauer‑)Diagnose Originaltext, ICD-10-Diagnosecodes, Diagnosesicherheit, Seitenlokalisation
*Abrechnungsziffern*	Gebührennummern, Gebührenordnungspositionen, Sach- und Materialkosten, Hausbesuche
*Verordnungen*	Medikamentenname, Pharmazentralnummer, ATC-Wirkstoffgruppe, Dosis, Preis, Dauerverordnung
*Inanspruchnahme*	Überweisungen, Arbeitsunfähigkeitsbescheinigungen, Hospitalisierung
**Exportart pseudonym**	
*Befunde*^a^	Laborbefund, Fremdbefund, Röntgenbefund, Symptome
*Risikofaktoren*^a^	Allergien, Unfälle, Operationen
*Therapieangaben*^a^	Verordnete Therapien
*Erhebungsinstrumente*	Studienspezifische Erhebungsinstrumente
Daten, die nicht erhoben werden	Soziale und ökonomische Daten (Gehalt, Familienstatus, Arbeitsstatus)

*ATC* anatomisch-therapeutisch-chemisches Klassifikationssystem der Weltgesundheitsorganisation, *ICD* Internationale statistische Klassifikation der Krankheiten und verwandter Gesundheitsprobleme

^a^Bis zur Freigabe der Anonymisierungssoftware werden diese Variablen nicht exportiert

Weiterhin wird ein Praxisprofil für administrative Zwecke und für eine spätere Validierung der Daten innerhalb des Projekts gespeichert. Das Profil umfasst den Namen der Praxis, die Betriebsstättennummer und die dazu gehörige lebenslang vergebene Arztnummer sowie die Personalausstattung.

### Aktueller Datenbestand

Aktuell übermitteln 40 Ärzte aus 5 Arztpraxen (inkl. 3 medizinische Versorgungszentren) mit 4 verschiedenen PVS monatlich Daten an die Datenbank. Es sind aktuell anonymisierte Daten von 73.043 Patienten (191.048 Patientenjahre) in der Datenbank vorhanden. Die Datenbank zählt 2.653.437 nach ICD-10 kodierten Diagnosen, die 6182 unterschiedlichen ICD-Diagnosecodes zugeordnet werden. Weiterhin sind Angaben zu 1.403.726 Verordnungen und 1.894.074 Laborergebnissen enthalten. Es existieren Patientendaten zu 6176 Patienten mit Diabetes mellitus Typ 2, 17.963 Patienten mit Hypertonie und 2911 Patienten mit Depression. Von 481 Patienten, die eingewilligt hatten, wurden die Daten erfolgreich pseudonymisiert exportiert.

Die Datenbank kann unmittelbar zur Testung von Hypothesen im Rahmen von Beobachtungsstudien verwendet werden und beispielweise für (pharmako)epidemiologische Studien und Studien innerhalb der Versorgungsforschung eingesetzt werden. Anonymisierte Feedbackberichte z. B. zu den häufigsten verschriebenen ATC-Wirkstoffen auf Praxisebene im Vergleich zum gesamten Praxispanel konnten für die teilnehmenden Praxen generiert werden.

Zur Überprüfung der Anonymisierung wurde eine manuelle Analyse der ersten 25.103 Patientendatensätze durch 2 Mitarbeiter in 2 Praxen vor Ort vorgenommen, aus denen Daten exportiert werden sollten. Im 4‑Augen-Prinzip wurden von allen Freitextfeldern mit dem Dateityp „varchar“ („variable character“) willkürlich je 50 Einträge analysiert, bei denen potenziell identifizierende Merkmale auftreten können. Es wurden keine identifizierenden Merkmale gefunden.

## Vorteile des BeoNet-Halle

Die ersten Versuche der Datenintegration zeigen, dass technische und datenschutzrechtliche Hürden bei der Datenextraktion überwunden und die Daten erfolgreich in die Datenbank überführt werden konnten. Die aggregierten, durch das BeoNet-Halle gesammelten Daten sollen auf lange Sicht Prozesse im Versorgungsalltag optimieren und wichtige Informationen zum Versorgungsgeschehen in teilnehmenden Praxen liefern. Wirksame Mittel zur Prozessoptimierung können bspw. periodische aktuelle Berichte zu Verschreibungs- und Krankheitsmustern und zur Inanspruchnahme von Versorgungsleistungen sein. An der konkreten Umsetzung eines vollautomatisierten Berichtswesens wird zurzeit gearbeitet.

Ein wichtiger Vorteil gegenüber Abrechnungsdaten sind die Extraktion und Zusammenführung von klinisch relevanten Informationen wie Laborergebnissen, BMI, Rauch- und Schwangerschaftsstatus etc. Theoretisch können mit dem Exportmodul sämtliche elektronische Informationen lückenlos aus PVS, einschließlich Arztbriefe, medizinische Bilder etc., extrahiert werden.

Sowohl prospektive als auch retrospektive Beobachtungsstudien werden durch das BeoNet-Projekt in ihrer Durchführbarkeit deutlich vereinfacht, vor allem da der Prozess der Rekrutierung problemlos in den Regelbetrieb einer Arztpraxis integriert werden kann. Zudem wird der Prozess der Studienbegleitung und der Datenerhebung erheblich vereinfacht, u. a. da studienspezifische Informationen direkt in das PVS eingegeben und durch das Exportmodul ausgelesen werden können. Weil mit Einwilligungen gearbeitet wird und nicht gesondert über die Bewertung von Daten z. B. im Rahmen einer Beobachtungsstudie informiert werden muss, ist das Auftreten von Verzerrungseffekten zudem weniger wahrscheinlich. Dies setzt allerdings voraus, dass die BeoNet-Halle-Einwilligungen von Patienten unverzerrt gegeben werden. Mitarbeiter analysieren aktuell im Rahmen einer deskriptiven Studie, inwiefern sozioökonomische Verzerrungen bei der Einholung von Einwilligungen auftreten.

Arztpraxen leisten mit der Teilnahme am BeoNet-Halle einen wichtigen Beitrag zur evidenzbasierten Medizin, u. a. durch die Beteiligung an Forschungsprojekten. In einem ersten Schritt erhalten teilnehmende Praxen für die Einholung von Einwilligungen eine Aufwandsentschädigung i. H. v. 2,00 € pro unterschriebener Einwilligung.

Vor der Entwicklung der Datenbank wurde intensiv nach länderspezifischen Barrieren und vergangenen Projekterfolgen und -misserfolgen recherchiert, die dem Aufbau einer repräsentativen ambulanten Datenbank in Deutschland entgegenstehen könnten. Zu den größten Herausforderungen, die von den Projekten berichtet wurden, gehören geringe Rekrutierungsraten, die Integration des Prozesses der Datenextraktion in den Praxisalltag, Schwierigkeiten bei der Extraktion von Daten aus unterschiedlichen PVS, die sektorenübergreifende Verknüpfung von Daten und Finanzierungsschwierigkeiten [9, 28–30]. Die uneinheitliche Realisierung der BDT-Schnittstelle seitens der Softwarehäuser stellte die Forscher vor große Herausforderungen hinsichtlich der Datenbereinigung und -auswertung. Die für unser Projekt entwickelten Exportmodule scheinen hier eine sinnvolle Ergänzung zu sein. Unser Kooperationspartner hat bereits Konverter für mehr als 70 verschiedene PVS entwickelt, mit denen Daten über mehrere Praxen hinweg mit gleichbleibender Qualität exportiert werden können.

Zusätzlich ging in vorangegangenen Studien der Export mit einem erheblichen Zeitaufwand für teilnehmende Arztpraxen einher, weil die BDT-Schnittstelle teilweise manuell freigeschaltet werden musste. Im BeoNet-Halle hingegen ist der Aufwand für die Praxis, unabhängig vom PVS, vergleichsweise gering und umfasst bei der anonymisierten Exportvariante lediglich die einmalige Installation des Exportmoduls und die regelmäßige Einspielung von Updates. Beim pseudonymisierten Export kann die Eingabe des Kürzels zum Einwilligungs- und Widerrufstatus mühelos im Regelbetrieb vorgenommen werden.

## Grenzen der Datenbank

Bei der Nutzung der BeoNet-Halle-Datenbank für Forschungszwecke ist die korrekte Eingabe in das PVS durch den behandelnden Arzt von entscheidender Bedeutung. Dabei spielen primär durch Menschen verursachte Eingabefehler oder das Weglassen von Angaben eine zentrale Rolle. Systematische Fehler bei der Dokumentation können zu Bias führen, die vorwiegend bei bestimmten Eingabemustern vorkommen können. Clegg et al. (2016) verwendete bspw. Informationen von geriatrischen Patienten aus einer englischen ambulanten Datenbank zur Entwicklung eines Frailty Index („frailty“, engl. für Gebrechlichkeit). Er fand heraus, dass die Daten mit hoher Wahrscheinlichkeit selektiv für gebrechlichere Patienten erfasst wurden [31, 32].

Bezogen auf den einzelnen Patienten ist eine sektorenübergreifende Beobachtung entlang aller möglichen Ebenen und Fachgebiete zurzeit nicht möglich. Beim Auftreten von notfallmedizinischen Erkrankungen, wie bspw. Herzinfarkten oder schwerwiegender Hypoglykämie, ist davon auszugehen, dass die Behandlung in einer Notfalleinrichtung erfolgt. Weiterhin ist beim Wechsel eines Patienten zu einer Praxis, die keine Daten an das BeoNet-Halle übermittelt, mit einem möglichen Bias bei der Berechnung des Follow-up zu rechnen. Dies ist eine Hauptlimitation für die BeoNet-Halle-Datenbank, die vor allem hinsichtlich der Durchführung von Studien zur Arzneimittelsicherheit berücksichtigt werden sollte. Daher eignen sich die BeoNet-Halle-Daten z. B. nicht zur Ableitung von Prävalenzdaten außerhalb der Haus- und Facharztpraxis.

Soziale und sozioökonomische Daten (Gehalt, Familienstatus, Berufsstatus, Bildung) werden aktuell nicht in der Datenbank abgebildet. Zudem enthalten die gegenwärtig verfügbaren klinischen Daten unvollständige Informationen zu Confoundern wie dem Rauchstatus, Anzahl der Kinder, Schwangerschaftsstatus, Größe, Gewicht, Blutdruckmessungen etc. Inwieweit diese Informationen überhaupt von den Ärzten in PVS erfasst werden, muss noch erforscht werden. In Zukunft werden mit kooperierenden Ärzten erstmalig Anstrengungen unternommen, um einige dieser Faktoren systematischer zu erheben und insgesamt die Dokumentationsqualität von in PVS eingegebenen Daten zu erhöhen.

## Ausblick

Die Ergebnisse des Projekts sind bisher vielversprechend und im nächsten Schritt wird eine vollwertige ambulante Datenbank mit mindestens 350.000 anonymisierten und 10.000 pseudonymisierten Datensätzen angestrebt. Mit dem BeoNet-Halle soll eine qualitativ hochwertige und für die Region Halle (Saale) und Umkreis repräsentative ambulante Datenbank geschaffen werden, die Modellcharakter besitzt und sich grundlegend zur Verknüpfung mit Daten von teilnehmenden Haus- und Fachärzten und langfristig auch zur Verknüpfung mit Daten von anderen Akteuren im Gesundheitswesen eignet. Besonders im Rahmen von Einwilligungen und der Bereitstellung der Infrastruktur der THS arbeiten wir eng zusammen mit dem MII-geförderten DIZ der UMH. Hierbei ist jedoch zu betonen, dass das BeoNet-Halle nicht der Governance der MII unterliegt. Die Ablage von Einwilligungen auf einer zentralen Instanz der THS, die Verwaltung und Zusammenführung von Daten werden hierbei mittels zertifizierter Instrumente der Universitätsmedizin Greifswald realisiert [11, 27]. Langfristig sollen die Einwilligungen auch elektronisch eingelesen werden und es wird eine Datenintegration auf Basis des Schnittstellen-Standards „H7 FHIR“ (Health Level 7 Fast Healthcare Interoperability Resources) angestrebt. Die Zusammenarbeit mit dem DIZ des UMH wird auch deshalb forciert, weil in der kommenden Förderperiode der MII der Austausch und die Nutzung von ambulanten Versorgungsdaten angestrebt werden.

Insbesondere im stationären Sektor, beispielsweise im Rahmen des Projektes Informationstechnische Systeme in Krankenhäusern (ISiK), wird aktuell die Forschung mit Versorgungsdaten vorangetrieben. Hier werden bereits FHIR-Schnittstellen-Standards erarbeitet [33]. Die Entwicklung von Schnittstellen erfordert eine enge Kooperation mit verschiedenen Akteuren des Gesundheitswesens. Im ambulanten Sektor wurde seitens des Gesetzgebers im Juni 2021 zwar die „PVS-Archivierungs- und Wechselschnittstelle“ auf Basis von FHIR vorgeschrieben, jedoch eignen sich die Datenexporte bislang nicht zum Aufbau einer derartigen Datenbank [34]. Daher stellt das hier vorgestellte Projekt eine sinnvolle Ergänzung zur Forschung mit ambulanten Versorgungsdaten dar.

Abschließend ist ein wichtiges Ziel vom BeoNet-Halle, kooperative wissenschaftliche Netzwerke mit wissenschaftlichem Personal und externen Forschern aufzubauen. Es ist angedacht, dass externe Forscher oder auch teilnehmende Praxen neben monatlichen Feedback-Berichten zur Inanspruchnahme und dem Verschreibungsverhalten Zugang zu Datenbankansichten erhalten sollen. Hierfür soll auf der Homepage ein geschützter Bereich eingerichtet werden.

## Supplementary Information





## Footnotes

1 Link zur Website: https://webszh.uk-halle.de/beonet/.

2 ATC: anatomisch-therapeutisch-chemisches Klassifikationssystem der Weltgesundheitsorganisation (WHO), eine internationale Klassifikation für Arzneimittelwirkstoffe.
