# Supplementary material for: BeoNet-Halle—development of a multifunctional database for the automated extraction of healthcare data from general practitioner and specialist practices
Source: Bundesgesundheitsblatt Gesundheitsforschung Gesundheitsschutz. 2023 Apr 20;66(5):569–77. [Article in German] doi: 10.1007/s00103-023-03691-7 (PMC10163113; doi:10.1007/s00103-023-03691-7)
Supplement: Supplementary file 1 [file 103_2023_3691_MOESM1_ESM.pdf]

Tabellenstruktur BeoNet-Halle

Inhaltsverzeichnis

Tabelle Praxis.csv 2
Tabelle Einwilligung.csv 2
Tabelle Stammdaten.csv 3
Tabelle Arztkontakt.csv 4
Tabelle Risiko.csv 4
Tabelle KlinischeParameter.csv 5
Tabelle Befunde.csv 6
Tabelle Labor.csv 7
Tabelle Diagnosen.csv 9
Tabelle Leistungsziffern.csv 9
Tabelle Medikamente.csv 11
Tabelle Therapie.csv 12
Tabelle Bescheinigung.csv 13

Tabelle Praxis.csv

	Feld	Exp	Typ	Länge	Inhalt
1	PraxisKey	PA	C		eindeutiger Key auf die gesamte Praxis
2	ArztKey	PA	C		eindeutiger Key auf einen Arzt
3	BSNR	PA	N	9	Betriebstättennummer
4	Betriebsstätte	PA	C		Bezeichnung der Betriebsstätte
5	Straße	PA	C		Straße der BS
6	Plz	PA	C	≤7	Postleitzahl der BS
7	Ort	PA	C		Ort der BS
8	LANR	PA	N	9	Lebenslange Arztnummer
9	Arzt	PA	C		Name des Arztes
10	Fachgruppe	PA	N	2	Fachrichtung des Arztes

Tabelle Einwilligung.csv

	Feld	Exp	Typ	Länge	Inhalt
1	PSPatientKey	P	C		eindeutiger Patientenkey
2	EinwilligungDatum	P	D	10	Datum der Statusänderung
3	EinwilligungStatus	P	S		Einwilligungsstatus

Tabelle Stammdaten.csv

	Feld	Exp	Typ	Länge	Inhalt
1	PSPatientKey	P	C		eindeutiger Patientenkey praxisübergreifend zuordenbar
2	ANPatientKey	PA	C		eindeutiger Patientenkey nur praxisintern und nur innerhalb eines Exportes eindeutig
3	Geburt	PA	C	7	Geburtsjahr +Monat des Patienten
4	Plz	PA	C	3	Postleitzahl des Patienten
5	VersichertenArt	PA	S	1	Versichertenart des Patienten
6	Geschlecht	PA	S	1	Geschlecht des Patienten
7	ErsterKontakt	PA	D	10	erster Arztkontakt im akt. Zeitraum
8	LetzterKontakt	PA	D	10	letzter Arztkontakt im akt. Zeitraum
9	KontakteGesamt	PA	N		Anzahl der Arztkontakte im akt. Zeitraum
10	Nationalität	PA	C	≤20	Nationalität des Patienten
11	AnzahlKind	PA	N	≤3	Anzahl Kinder
12	AnzahlSchwanger	PA	N	≤3	Anzahl Schwangerschaften
13	RaucherAnamnese	PA	S	≤1	
14	RaucherTyp	PA	S	≤2	
15	PraxisKey	PA	C		eindeutiger Praxiskey
16	ArztKey	PA	C		eindeutiger Arztkey

Tabelle Arztkontakt.csv

	Feld	Exp	Typ	Länge	Inhalt
1	KontaktID	PA	N		lfd. Nummerierung der Kontakte
2	Datum	PA	D	10	Behandlungsdatum
3	PSPatientKey	P	C		eindeutiger Patientenkey
4	ANPatientKey	PA	C		eindeutiger Patientenkey
5	Fachgruppe	PA	N	2	Fachrichtung des Arztes
6	PraxisKey	PA	C		eindeutiger Praxiskey
7	ArztKey	PA	C		eindeutiger Arztkey

Tabelle Risiko.csv[1]

	Feld	Exp	Typ	Länge	Inhalt
1	Risiko_KID	P[1]	N		Arztkontakt-ID
2	Allergie	P[1]	C		Allergien
3	Unfall	P[1]	C		Unfälle
4	Operation	P[1]	C		Operationen
5	Anamnese	P[1]	C		Anamnesen
6	Risiko	P[1]	C		Risikofaktoren (Cave)
7	PSPatientKey	P	C		eindeutiger Patientenkey
8	ANPatientKey	P	C		eindeutiger Patientenkey
9	Risiko_Datum	P	D	10	Datum
10	Fachgruppe	P	N	2	Fachrichtung des Arztes
11	PraxisKey	P	C		eindeutiger Praxiskey
12	ArztKey	P	C		eindeutiger Arztkey

Tabelle KlinischeParameter.csv

	Feld	Exp	Typ	Länge	Inhalt
1	KP_KID	PA	N		Arztkontakt-ID
2	Größe	PA	N	≤3	Körpergröße in cm
3	Gewicht	PA	N	≤3	Gewicht in kg
4	Temperatur	PA	N	≤2	Körpertemperatur in °C
5	HueftUmfang	PA	N	≤3	Hüftumfang in cm
6	BauchUmfang	PA	N	≤3	Bauchumfang in cm
7	KopfUmfang	PA	N	≤3	Kopfumfang in cm
8	Blutdruck	PA	C	≤7	Blutdruck
9	Puls	PA	N	≤3	Puls in min-1
10	PSPatientKey	P	C		eindeutiger Patientenkey
11	ANPatientKey	PA	C		eindeutiger Patientenkey
12	KP_Datum	PA	D	10	Datum
13	Fachgruppe	PA	N	2	Fachrichtung des Arztes
14	PraxisKey	PA	C		eindeutiger Praxiskey
15	ArztKey	PA	C		eindeutiger Arztkey

Tabelle Befunde.csv[1]

	Feld	Exp	Typ	Länge	Inhalt
1	Befunde_KID	P[1]	N		Arztkontakt-ID
2	Befund	P[1]	C		Befunde
3	FremdBefund	P[1]	C		Fremdbefunde
4	LaborBefund	P[1]	C		Laborbefunde
5	RoentgenBefund	P[1]	C		Röntgenbefunde
6	Symptom	P[1]	C		Symptome
7	PSPatientKey	P	C		eindeutiger Patientenkey
8	ANPatientKey	P	C		eindeutiger Patientenkey
9	Befunde_Datum	P	D	10	Datum
10	Fachgruppe	P	N	2	Fachrichtung des Arztes
11	PraxisKey	P	C		eindeutiger Praxiskey
12	ArztKey	P	C		eindeutiger Arztkey

Tabelle Labor.csv

	Feld	Exp	Typ	Länge	Inhalt
1	Labor_KID	PA	N		Arztkontakt-ID
2	BefundArt	PA	S	1	Befundart
3	TestIdent	PA	C		
4	TestBezeichnung	PA	C		
5	TestStatus	PA	S	1	
6	ErgebnisWert	PA	N		Ergebnis – Wert
7	ErgebnisText	PA	C	≤60	Ergebnis – Text
8	Einheit	PA	C	≤60	
9	Grenzwert	PA	S	≤2	Grenzwertindikator
10	ProbenMatIdent	PA	C	≤60	Probenmaterial-Ident
11	ProbenMatIndex	PA	N		Probenmaterial-Index
12	ProbenMatBezeichnung	PA	C	≤60	Probenmaterial-Bezeichnung
13	ProbenMatMenge	PA	C	≤60	Menge des Probenmaterials
14	ProbenMatEinheit	PA	C	≤60	Einheit des Probenmaterials
15	MatSpezifikation	PA	C	≤60	Material-Spezifikation
16	AbnahmeDatum	PA	D	10	Abnahme-Datum
17	AbnahmeZeit	PA	C	8	Abnahme-Zeit
18	KeimIdent	PA	C	≤60	Keim-Ident
19	KeimBezeichnung	PA	C	≤60	Keim-Bezeichnung
20	KeimNummer	PA	C	≤60	Keim-Nummer

	Feld	Exp	Typ	Länge	Inhalt
21	ResistenzMethode	PA	S	1	Resistenz-Methode
22	ResistenzInterpretation	PA	C	≤60	Resistenz-Interpretation
23	WirkstoffIdent	PA	C	≤60	Wirkstoff-Ident
24	WirkstoffGenericNummer	PA	C	≤60	Wirkstoff-Generic-Nummer
25	MHKBreakpointWert	PA	C	≤60	MHK/Breakpoint-Wert
26	NormalwertText	PA	C	≤60	Normalwert-Text
27	NormalwertUntergrenze	PA	N		Normalwert-Untergrenze
28	NormalwertObergrenze	PA	N		Normalwert-Obergrenze
29	TestHinweis	P[1]	C		testbezogene Hinweise
30	AuftragHinweis	P[1]	C		auftragsbezogene Hinweise
31	Signatur	P[1]	C		Signatur
32	PSPatientKey	P	C		eindeutiger Patientenkey
33	ANPatientKey	PA	C		eindeutiger Patientenkey
34	Labor_Datum	PA	D	10	Datum
35	Fachgruppe	PA	N	2	Fachrichtung des Arztes
36	PraxisKey	PA	C		eindeutiger Praxiskey
37	ArztKey	PA	C		eindeutiger Arztkey

Tabelle Diagnosen.csv

	Feld	Exp	Typ	Länge	Inhalt
1	Diagnose_KID	PA	N		Arztkontakt-ID
2	DauerDiagnose	PA	N	1	1 = Dauerdiagnose
3	DauerSeit	PA	D	10	Dauerdiagnose seit
4	Diagnose	PA	C		Diagnose
5	ICD	PA	C	≤6	ICD-Schlüssel
6	ICDSicher	PA	S	1	Diagnosensicherheit
7	ICDSeite	PA	S	1	Seitenlokalisation
8	Erlaeuterung	P	C		Diagnosenerläuterung
9	Ausnahme	P	C		Diagnosenausnahmetatbestand
10	PSPatientKey	P	C		eindeutiger Patientenkey
11	ANPatientKey	PA	C		eindeutiger Patientenkey
12	Diagnose_Datum	PA	D	10	Datum
13	Fachgruppe	PA	N	2	Fachrichtung des Arztes
14	PraxisKey	PA	C		eindeutiger Praxiskey
15	ArztKey	PA	C		eindeutiger Arztkey

Tabelle Leistungsziffern.csv

	Feld	Exp	Typ	Länge	Inhalt
1	Ziffer_KID	PA	N		Arztkontakt-ID
2	Ziffer	PA	C	≤ 9	Leistungsziffer

	Feld	Exp	Typ	Länge	Inhalt
3	ZifferTyp	PA	S	1	Abrechnungstyp
4	Untersuchungsart	PA	C	≤60	FK 5002
5	SachkostenName	PA	C	≤60	FK 5011
6	SachkostenBetrag	PA	N	≤ 10	FK 5012
7	HerstellerName	PA	C	≤60	FK 5074
8	Organ	PA	C	≤60	FK 5015
9	Hausbesuch	PA	S	1	(FK 5017)
10	Wiederholung	PA	S	1	FK 5020
11	Krebsvorsorge	PA	N	4	FK 5021
12	PostStationaer	PA	S	1	FK 5024
13	AufnahmeDatum	PA	D	10	FK 5025
14	EntlassungsDatum	PA	D	10	FK 5026
15	OPDatum	PA	D	10	FK 5034
16	OPSchluessel	PA	C		FK 5035
17	Komplikation	P[1]	C		FK 5038
18	GenName	PA	C	≤60	FK 5072
19	Erkrankungsart	PA	C	≤60	FK 5073
20	PSPatientKey	P	C		eindeutiger Patientenkey
21	ANPatientKey	PA	C		eindeutiger Patientenkey
22	Ziffer_Datum	PA	D	10	Datum
23	Fachgruppe	PA	N	2	Fachrichtung des Arztes
24	PraxisKey	PA	C		eindeutiger Praxiskey
25	ArztKey	PA	C		eindeutiger Arztkey

Tabelle Medikamente.csv

	Feld	Exp	Typ	Länge	Inhalt
1	Medikamente_KID	PA	N		Arztkontakt-ID
2	VOTyp	PA	S	≤2	Typ der Verordnung
3	PZN	PA	N		Pharmazentralnummer
4	Medikamentenname	PA	C		Medikamentenname
5	ATCCode	PA	C		ATC-Code
6	ATCWirkstoffname	PA	C		ATC-Wirkstoffname
7	Preis	PA	N		Preis
8	MedTyp	PA	S	1	Medikamententyp
9	VerordnungsArt	PA	S	1	
10	GebuehrenPflicht	PA	S	1	gebührenpflichtig
11	AutIdem	PA	S	1	Aut Idem
12	PackungZahl	PA	N	≤3	Anzahl Packungen
13	Einnahme	PA	C	<20	Einnahmeplan
14	Zusatz	P	C		Zusätze
15	DauerMed	PA	S	1	Dauermedikament
16	Abgesetzt	PA	D	10	Dauermed. abgesetzt am
17	PSPatientKey	P	C		eindeutiger Patientenkey
18	ANPatientKey	PA	C		eindeutiger Patientenkey
19	Medikamente_Datum	PA	D	10	Datum
20	Fachgruppe	PA	N	2	Fachrichtung des Arztes
21	PraxisKey	PA	C		eindeutiger Praxiskey
22	ArztKey	PA	C		eindeutiger Arztkey

Tabelle Therapie.csv

	Feld	Exp	Typ	Länge	Inhalt
1	Therapie_KID	PA	N		Arztkontakt-ID
2	Therapie	P[1]	C		Therapien
3	PhysTherapie	P[1]	C		phys. Therapien
4	UeberweisungAn	PA	C	≤60	Überweisung an
5	UeberweisungText	P[1]	C		Inhalt der Überweisung
6	AUVon	PA	D	10	arbeitsunfähig ab
7	AUBis	PA	D	10	arbeitsunfähig bis
8	AUGrund	P[1]	C		Grund der Arbeitsunfähigkeit
9	EinweisungAn	PA	C	≤60	Krankenhauseinweisung: Krankenhaus
10	EinweisungGrund	P[1]	C		Grund der Krankenhauseinweisung
11	PSPatientKey	P	C		eindeutiger Patientenkey
12	ANPatientKey	PA	C		eindeutiger Patientenkey
13	Therapie_Datum	PA	D	10	Datum
14	Fachgruppe	PA	N	2	Fachrichtung des Arztes
15	PraxisKey	PA	C		eindeutiger Praxiskey
16	ArztKey	PA	C		eindeutiger Arztkey

Tabelle Bescheinigung.csv[1]

	Feld	Exp	Typ	Länge	Inhalt
1	Bescheinigung_KID	P[1]	N		Arztkontakt-ID
2	Typ	P[1]	C		Typ der Bescheinigung
3	Inhalt	P[1]	C		Inhalt der Bescheinigung
4	Link	P[1]	C		Verweis bei Bildern, Briefen, Links
5	PSPatientKey	P	C		eindeutiger Patientenkey
6	ANPatientKey	P	C		eindeutiger Patientenkey
7	Bescheinigung_Datum	P	D	10	Datum
8	Fachgruppe	P	N	2	Fachrichtung des Arztes
9	PraxisKey	P	C		eindeutiger Praxiskey
10	ArztKey	P	C		eindeutiger Arztkey

Bilder, Briefe, Links:

Typ = „Bild", „Brief", „Link"
Inhalt = „Arztbrief", „Sonografie des Bauches", …
Link = Verweis auf die Datei/URL

Feldtypen:

C Varchar
S fester Eintrag aus einer Menge von möglichen Werten (bzw. leer)
N nummerischer Eintrag
D Datum im Format jjjj-mm-tt

Exportart (Exp):

A nur bei anonymisiertem Export
P nur bei pseudonymisiertem Export
PA immer

[1] Bis zur Freigabe des Anonymisierungstools bleiben die kompletten Tabellen bzw. einige Felder leer.
